# Re-irradiation of recurrent glioblastoma multiforme using ^11^C-methionine PET/CT/MRI image fusion for hypofractionated stereotactic radiotherapy by intensity modulated radiation therapy

**DOI:** 10.1186/1748-717X-9-181

**Published:** 2014-08-14

**Authors:** Kazuhiro Miwa, Masayuki Matsuo, Shin-ichi Ogawa, Jun Shinoda, Kazutoshi Yokoyama, Jitsuhiro Yamada, Hirohito Yano, Toru Iwama

**Affiliations:** Chubu Medical Center for Prolonged Traumatic Brain Dysfunction, Kizawa Memorial Hospital, Minokamo, Gifu, Japan; Department of Radiation Oncology, Kizawa Memorial Hospital, Minokamo, Gifu, Japan; Department of Neurosurgery, Kizawa Memorial Hospital, Minokamo, Gifu, Japan; Department of Neurosurgery, Gifu University Graduate School of Medicine, Gifu, Japan; Department of Neurosurgery, Chubu Medical Center for Prolonged Traumatic Brain Dysfunction, 630 Shimokobi, Kobi-cho, Minokamo, Gifu, 505-0034 Japan

**Keywords:** Recurrent glioblastoma multiforme, Hypofractionated stereotactic radiotherapy, Intensity modulated radiation therapy, ^11^C-methionine PET

## Abstract

**Background:**

This research paper presents a valid treatment strategy for recurrent glioblastoma multiforme (GBM) using hypofractionated stereotactic radiotherapy by intensity modulated radiation therapy (HS-IMRT) planned with ^11^C-methionine positron emission tomography (MET-PET)/computed tomography (CT)/magnetic resonance imaging (MRI) fusion.

**Methods:**

Twenty-one patients with recurrent GBM received HS-IMRT planned by MET-PET/CT/MRI. The region of increased amino acid tracer uptake on MET-PET was defined as the gross tumor volume (GTV). The planning target volume encompassed the GTV by a 3-mm margin. Treatment was performed with a total dose of 25- to 35-Gy, given as 5- to 7-Gy daily for 5 days.

**Results:**

With a median follow-up of 12 months, median overall survival time (OS) was 11 months from the start of HS-IMRT, with a 6-month and 1-year survival rate of 71.4% and 38.1%, respectively. Karnofsky performance status was a significant prognostic factor of OS as tested by univariate and multivariate analysis. Re-operation rate was 4.8% for radiation necrosis. No other acute or late toxicity Grade 3 or higher was observed.

**Conclusions:**

This is the first prospective study of biologic imaging optimized HS-IMRT in recurrent GBM. HS-IMRT with PET data seems to be well tolerated and resulted in a median survival time of 11 months after HS-IMRT.

## Background

In recurrent gliomas retreated with radiation therapy, precise dose delivery is extremely important in order to reduce the risk of normal brain toxicity. Recently, novel treatment modalities with increased radiation dose-target conformality, such as intensity modulated radiation therapy (IMRT), have been introduced [1–4]. While IMRT has superior target isodose coverage compared to other external radiation techniques in scenarios involving geometrically complex target volumes adjacent to radiosensitive tissues, planning and delivery in IMRT are resource intensive and require specific and costly software and hardware.

Gross tumor volume (GTV) delineation in gliomas has been traditionally based on computed tomography (CT) and magnetic resonance imaging (MRI). However, ^11^C-methionine positron emission tomography (MET-PET) for high-grade gliomas was recently demonstrated to have an improved specificity and sensitivity and is the rationale for the integration of biologic imaging in the treatment planning [5–8]. In previous studies using MET-PET/MRI image fusion, we demonstrated that biologic imaging helps to detect tumor infiltration in regions with a non-specific MRI appearance in a significant number of patients [9–11]. Moreover, non-specific post-radiotherapeutic changes (e.g., radiation necrosis, gliosis, unspecific blood–brain barrier disturbance) could be differentiated from tumor tissue with a higher accuracy [12–14]. A recent study demonstrated that MET-PET could improve the ability to identify areas with a high risk of local failure in GBM patients [15].

Based on the prior PET studies, we hypothesized that an approach of hypofractionated stereotactic radiotherapy by IMRT (HS-IMRT) with the use of MET-PET data would be an effective strategy for recurrent GBM. This prospective study was designed to measure the acute and late toxicity of patients treated with HS-IMRT planned by MET-PET, response of recurrent GBM to this treatment, overall survival (OS), and the time to disease progression after treatment.

## Methods

### Patients eligibility

Patients were recruited from September 2007 to August 2011. Adult patients (aged ≥ 18 years) with histopathologic confirmation of GBM who had local recurrent tumor were eligible. Primary treatment consisted of subtotal surgical resection in all patients. All patients had a Karnofsky performance status (KPS) ≥ 60 and were previously treated with external postoperative radiotherapy to a mean and median dose of 60 Gy (range 54 Gy–68 Gy) with concomitant and adjuvant Temozolomide (TMZ) chemotherapy. Additional inclusion criteria consisted of the following: age 18 years or older; first macroscopical relapse at the original site; and adequate bone marrow, hepatic, and renal function. In all cases, a multidisciplinary panel judged the resectability of the lesion before inclusion in this study. Thus patients with non-resectable lesions were included in the study.

### Study design

This prospective nonrandomized single-institution study was approved by the Department of Radiation Oncology of Kizawa Memorial Hospital Institutional Review Board. Informed consent was obtained from each subject after disclosing the potential risks of HS-IMRT and discussion of potential alternative treatments. Baseline evaluation included gadolinium-enhanced brain MRI and MET-PET, complete physical and neurological examination, and blood and urine tests within 2 weeks before treatment. After completion of HS-IMRT, patients underwent a physical and neurological examination and a repeat brain MRI and MET-PET. TMZ chemotherapy was continued in a manner consistent with standard clinical use after HS-IMRT course.

### Imaging: CT

CT (matrix size: 512 × 512, FOV 50 × 50 cm) was performed using a helical CT instrument (Light Speed; General Electric, Waukesha, WI). The patient head was immobilized in a commercially available stereotactic mask, and scans were performed with a 2.5-mm slice thickness without a gap.

### Imaging: MRI

MRI (matrix size: 256 × 256, FOV 25 × 25 cm) for radiation treatment planning was performed using a 1.5-T instrument (Light Speed; General Electric). A standard head coil without rigid immobilization was used. An axial, three-dimensional gradient echo T1-weighted sequence with gadolinium and 2.0-mm slice thickness were acquired from the foramen magnum to the vertex, perpendicular to the main magnetic field.

### Imaging: MET-PET

An ADVANCE NXi Imaging System (General Electric Yokokawa Medical System, Hino-shi, Tokyo), which provides 35 transaxial images at 4.25-mm intervals, was used for PET scanning. A crystal width of 4.0 mm (transaxial) was used. The in-plane spatial resolution (full width at half-maximum) was 4.8 mm, and scans were performed in a standard two-dimensional mode. Before emission scans were performed, a 3-min transmission scan was performed to correct photon attenuation using a ring source containing 68Ge. A dose of 7.0 MBq/kg of MET was injected intravenously. Emission scans were acquired for 30 min, beginning 5 min after MET injection. During MET-PET data acquisition, head motion was continuously monitored using laser beams projected onto ink markers drawn over the forehead skin and corrected as necessary. PET/CT and MRI volumes were fused using commercially available software (Syntegra, Philips Medical System, Fitchburg, WI) using a combination of automatic and manual methods. Fusion accuracy was evaluated by a consensus of 3 expert radiologists with 15 years of experience using anatomical fiducials such as the eyeball, lacrimal glands, and lateral ventricles.

### Radiation technique

**Figure 1:**
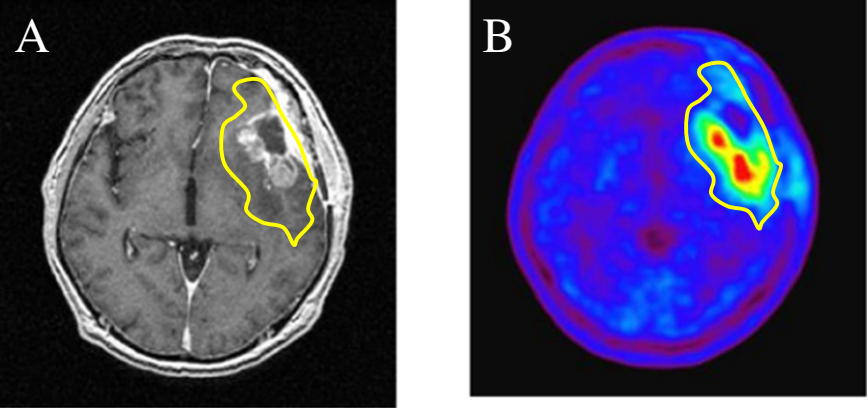
**An example of a target planned for a hypofractionated stereotactic radiotherapy using intensity modulated radiation therapy. (A)** Contrast-enhanced T1-weighted magnetic resonance imaging. **(B)** ^11^C-methionine positron emission tomography (MET-PET). Gross tumor volume was defined as the region with high MET uptake (yellow line). The threshold for increased MET uptake was set to ≥1.3 in the contiguous tumor region.

**Figure 2:**
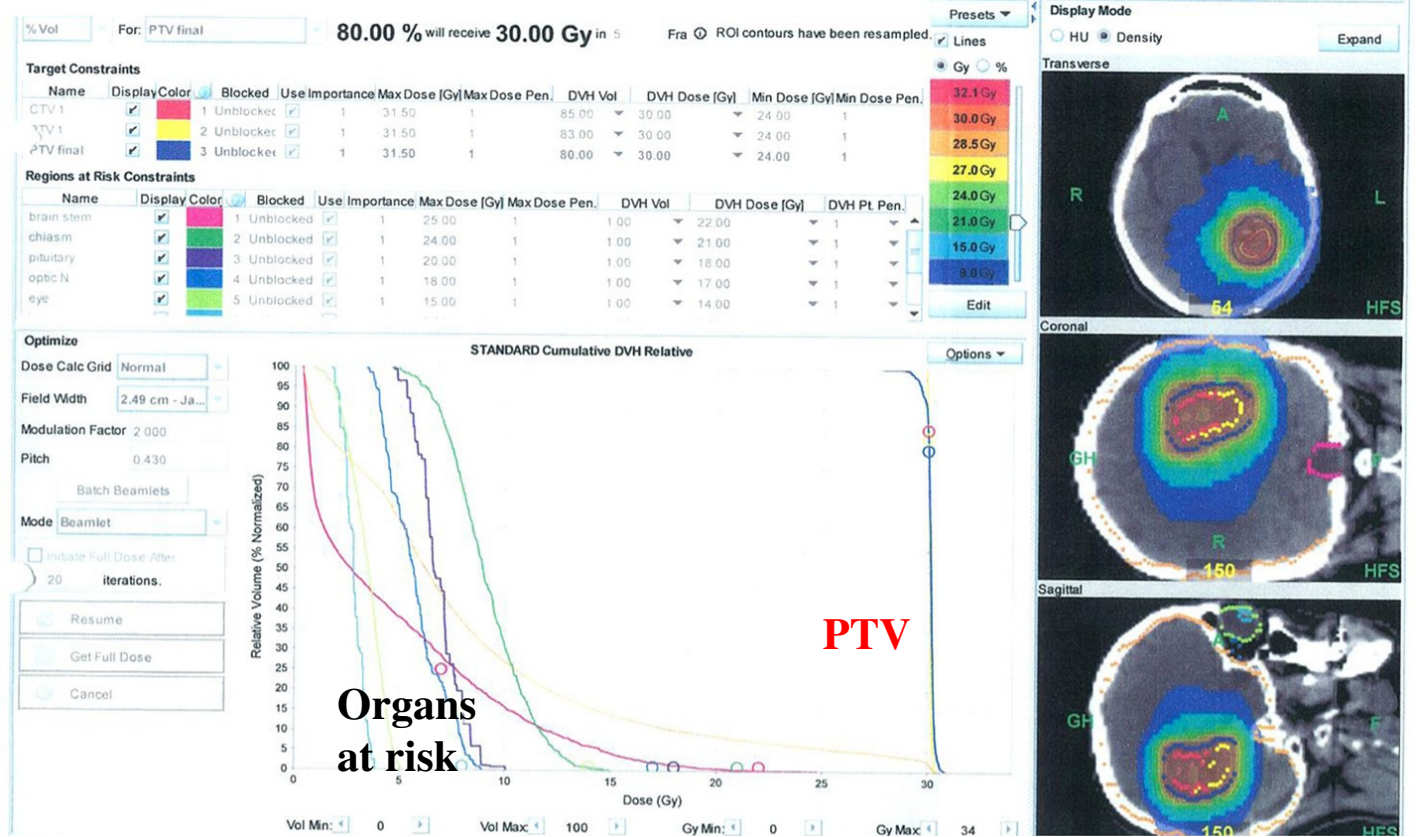
**A dose map and dose-volume histogram of a representative case.** Prescribed dose for planning target volume (PTV) and the doses of organs at risk were demonstrated.

Target volumes were delineated on the registered PET/CT-MRI images and were used to plan HS-IMRT delivery. In this preliminary study, GTV was defined as using an area of high uptake on MET-PET (Figure 1). This high-uptake region was defined using a threshold value for the lesion versus normal tissue counts of radioisotope per pixel index of at least 1.3. A previous study with high-grade gliomas, comparing the exact local MET uptake with histology of stereotaxically guided biopsies, demonstrated a sensitivity of 87% and specificity of 89% for the detection of tumor tissue at the same threshold of 1.3-fold MET uptake relative to normal brain tissue [16]. Further, this threshold was validated in a study using diffusion tensor imaging in comparison with MET uptake [17]. Although the delineation of GTV was done by using the automatic contouring mode (Philips Pinnacle v9.0 treatment planning system), the final determination of GTV was confirmed by consensus among 3 observers based on the co-registered MRI and PET data. The PET/MRI fusion image was positioned properly by CT scans equipped with Helical TomoTherapy (TomoTherapy Inc., Madison, WI). Finally, the GTV was expanded uniformly by 3 mm to generate the planning target volume (PTV). The prescribed dose for re-irradiation was based on tumor volume, prior radiation dose, time since external postoperative radiotherapy, and location of the lesion with proximity to eloquent brain or radiosensitive structures. Radiosensitive structures, including the brainstem, optic chiasm, lens, optic nerves, and cerebral cortex, were outlined, and dose-volume histograms for each structure were obtained to ensure that doses delivered to these structures were tolerable. Considering these different conditions, the dose for PTV was prescribed using the 80-95% isodose line, and the total doses were arranged from 25 Gy to 35 Gy in each patient. The dose maps and dose-volume histograms of a representative case are illustrated in Figure 2.

### Chemotherapy

After HS-IMRT course, a dose of 200 mg/m^2^/day for 5 days with TMZ chemotherapy was administered. Cycles were repeated every 28 days with 3 or more cycles after HS-IMRT. Treatment was discontinued when unequivocal progression or severe toxicity occurred. TMZ was not administrated in patients who refused treatment or did not meet inclusion criteria. Patient inclusion criteria for TMZ chemotherapy consisted of the following: KPS score 50 or higher; evidence of good maintenance of major organ function (bone marrow, liver, kidney, etc.) in routine laboratory studies; expected to survive more than 3 months.

### Follow-up

Regular serial neurological and radiological examinations were initially performed at 1 month after completion of treatment and then every 2 months thereafter or in the event of neurological decay. Follow-up examinations included MRI and MET-PET imaging. If the MRI revealed further enlargement of the enhanced mass, the lesion was diagnosed as “local progression”, and the day on which MRI first revealed lesion enlargement was defined as the date of progression. However, in cases with low MET uptake on PET in the MRI-enhanced lesion, the diagnosis was changed to “radiation necrosis”. The criterion of MET-PET differential diagnosis between local progression and radiation necrosis was utilized in a previous MET-PET study by Takenaka et al. [18]. However, all patients couldn’t be applied with PET examination in the follow up study. In cases without PET examination, diagnosis of radiation necrosis was based on pathologic examination or clinical course. Cases in which lesions showed spontaneous shrinkage or decreased in size during corticosteroid treatment were also defined as “radiation necrosis”. A diagnosis of “distant failure” was defined as the appearance of a new enhanced lesion distant from the original tumor site. Either local progression or distant failure was defined as disease progression. Acute and late toxicities were determined based on the Common Terminology Criteria for Advance Events (version 4).

### Statistical analysis

Survival events were defined as death from any cause for OS and as disease progression for progression-free survival (PFS). OS and PFS were analyzed from the date of HS-IMRT to the date of the documented event using the Kaplan-Meier method. Tumor- and therapy-related variables were tested for a possible correlation with survival, using the Log-rank test. Variables included age (≥50 vs. <50), KPS (≥70 vs. <70), and combined TMZ chemotherapy (“yes” vs. “no”). P-values of less than 0.05 were considered significant. Prognostic factors were further evaluated in a multivariate stepwise Cox regression analysis.

## Results

Twenty-one patients (18 men, 3 women) with histologically confirmed GBM were enrolled in this trial (Table 1). The median patient age was 53.9 years (range 22–76), and median KPS was 80 (range 60–90).

**Table 1:** **Patient characteristics**

Parameter	n (%)
Age, years	
≥50	13 (62)
<50	8 (38)
Gender	
Male	18 (86)
Female	3 (14)
KPS score	
≥70	16 (76)
<70	5 (24)
Combined TMZ chemotherapy	
Yes	13 (62)
No	8 (38)

*Abbreviations:* *KPS* Karnofsky performance status, *TMZ* temozolomide.

The median elapsed time between external postoperative radiotherapy and study enrollment was 12 months (range, 3–48 months). HS-IMRT was performed in 5 fractions, keeping a total dose for the PTV at 25 Gy in 6 patients, 30 Gy in 11 patients, and 35 Gy in 4 patients, given as 5- to 7-Gy daily over a period of 5 days. The average PTV was 27.4 ± 24.1 cm^3^ (3.4 - 102.9 cm^3^). All 21 patients completed the prescribed HS-IMRT course. Overall, thirteen patients (62%) received combined modality treatment with TMZ.

### Toxicity assessment

No patients demonstrated significant acute toxicity, and all patients were able to complete the prescribed radiation dose without interruption. In the late phase, Grade 2 radiation necrosis was observed in 1 patient, although the lesion was decreased in size during corticosteroid treatment. One patient who received a dose of radiation of 25.0 Gy, experienced Grade 4 radiation necrosis in the form of mental deterioration 4 months after HS-IMRT. In this case, a second necrotomy was required 5 months after HS-IMRT. Although after a second surgery, the patient’s clinical condition was stable for a long period, disease progression occurred 40 months after HS-IMRT, causing death. The actuarial reoperation rate was 4.8% for radiation necrosis.

### Outcomes

**Figure 3:**
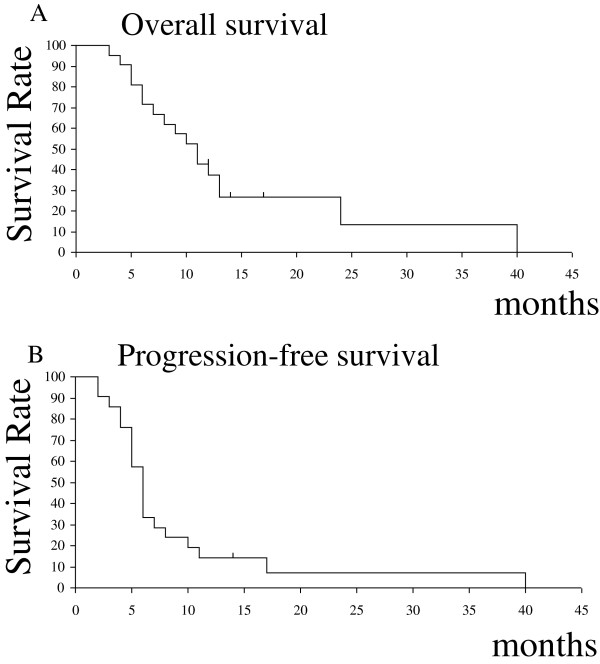
**(A) Overall survival and (B) Progression-free survival for all patients from the date of re-irradiation.** The median overall survival time was 11 months, and the median progression-free survival time was 6 months from the date of re-irradiation, respectively.

**Figure 4:**
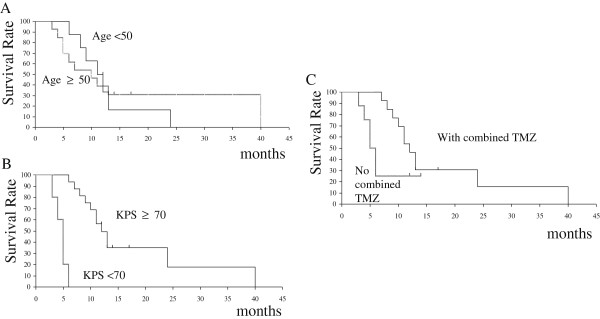
**Overall survival rates among different subgroups by (A) age, (B) Karnofsky performance status (KPS), and (C) combined temozolomide (TMZ) chemotherapy.**

With a median follow-up of 12 months, the median OS was 11 months from the date of HS-IMRT, with a 6-month and 1-year survival rate of 71.4% and 38.1%, respectively (Figure 3A). The survival rates by age, KPS, and TMZ chemotherapy are shown in Figure 4A-C. OS of patients with KPS 70 or over was 12 months versus 5 months for patients with KPS less than 70. KPS was a significant prognostic factor of OS as tested by univariate analysis (p < 0.001). OS of patients who received combined TMZ chemotherapy was 12 months versus 6 months for patients who did not receive chemotherapy. The differences between the two groups approached significance (p = 0.079). In the multivariate model, only KPS remained statistically significant (p = 0.005) (Table 2).

**Table 2:** **Analysis of prognostic variables for overall survival**

Variables	Median survival (months)	Univariate analysis* ***p***value	Multivariate analysis ^†^ ***p***value
Age			
≥50	10	0.931	
<50	11		
KPS			
≥70	12	<0.001	0.005
<70	5		
Combined TMZ chemotherapy			
Yes	12	0.079	0.581
No	6		

*Abbreviations* as in Table 1.

Statistical analyses were performed with *Log - rank test and ^†^Cox’s proportional hazards model.

The median PFS was 6 months from the date of HS-IMRT treatment, with a 6-month and 1-year survival rate of 38.1% and 14.3%, respectively (Figure 3B). KPS was the significant prognostic factor for PFS as tested by univariate analysis (p = 0.016). On multivariate analysis, none of the variables were significantly predictive of PFS (Table 3).

**Figure 5:**
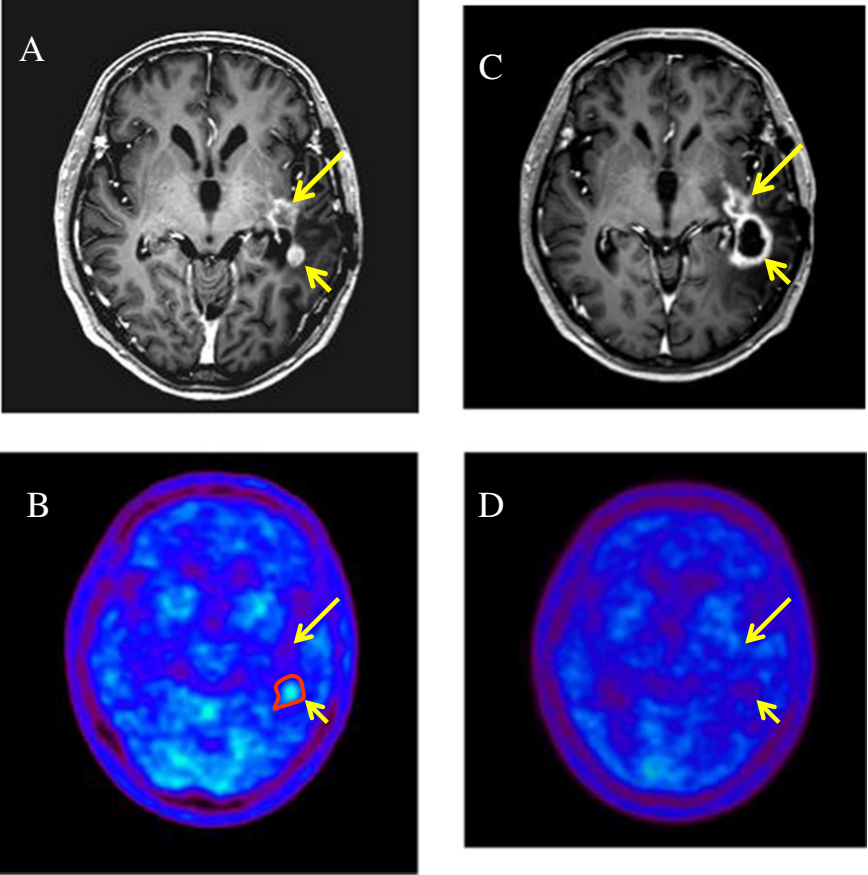
**Recurrent glioblastoma multiforme (GBM) in a 55-year-old man before and after hypofractionated stereotactic radiotherapy by intensity modulated radiation therapy (HS-IMRT).** Before HS-IMRT, two enhanced lesions (long and short arrow) were demonstrated in the left temporal lobe on T1-weighted magnetic resonance imaging **(A)**. ^11^C-methionine positron emission tomography (MET-PET) demonstrated a MET high-uptake on the region of short arrow, which was defined as the Gross Tumor Volume (red line) **(B)**. 5 months after HS-IMRT, there was no tumor recurrence on the lesion (long arrow, **C** &**D**). The enhanced lesion (short arrow) was increased in size **(C)**, although MET uptake decreased relative to normal tissue, which suggested a necrotic change in the irradiated region **(D)**. The patient had no neurologic deficits or quality of life issues. KPS was 90%.

A representative case is shown in Figure 5, in which a follow-up MET-PET scan was performed to improve the diagnostic accuracy.

**Table 3:** **Analysis of prognostic variables for progression - free survival**

Variables	Median survival (months)	Univariate analysis* ***p***value	Multivariate analysis ^†^ ***p***value
Age			
≥50	6	0.279	
<50	6		
KPS			
≥70	6	0.016	0.059
<70	5		
Combined TMZ chemotherapy			
Yes	6	0.447	0.479
No	5		

*Abbreviations* as in Table 1.

Statistical analyses were performed with *Log - rank test and ^†^Cox’s proportional hazards model.

## Discussion

Recently, much work has been performed on various fractionation regimens and dose escalation with IMRT for GBM. These studies reveal relatively favorable survival results [1–4], although a distinct advantage over conventional radiation therapy has not been demonstrated. However, if a precise conformal treatment technique such as HS-IMRT is utilized, and a greater biologic dose to the infiltrating tumor is achieved through hypo-fractionation, it could be possible to deliver an effective therapy that may increase patient survival without increasing morbidity. To meet these requirements, the contouring of the target volume is of critical importance.

PET is a newer imaging method that can improve the visualization of biological processes. In recent PET studies, analysis of the metabolic and histologic characteristics provided evidence that regional high MET uptake correlates with the malignant pathologic features [6–8, 16]. Furthermore, nonspecific post-therapeutic changes could be differentiated from tumor tissue with a higher accuracy [12–14]. These findings support the notion that complementary information derived from MET uptake may be helpful in developing individualized, patient-tailored therapy strategies in patients with recurrent GBM.

This prospective study was designed to measure the acute and late toxicity of patients treated with HS-IMRT planned by MET-PET, response of recurrent GBM to this treatment, OS, and the time to disease progression after treatment. We found that the OS rate from re-irradiation was 11 months and that the 6-month and 1-year OS rates were 71.4% and 38.1%, respectively (Figure 3). The survival results of our study appeared to be favorable compared to prior studies using other hypofractionated stereotactic radiotherapy in recurrent malignant glioma [19–22]. Also, we observed an improved toxicity profile, suggesting that hypofractionated stereotactic radiotherapy should be considered standard salvage therapy for previously irradiated high-grade gliomas [23]. We hypothesized that MET-PET/CT/MRI image fusion could facilitate target volume delineation and normal tissue sparing. This might lead to an improved therapeutic ratio, especially in pre-treated patients, in which post-therapy changes in CT or MRI, as with contrast enhancement, are difficult to distinguish from tumor recurrence (Figure 5).

In our study, KPS ≥ 70% was associated with longer patient survival, as previously described for recurrent GBM [21]. In addition, we observed a trend toward a beneficial effect on OS when combining TMZ chemotherapy (Figure 4C, Table 2). We estimated that the addition of TMZ might be particularly effective if the radiation dose to normal brain tissue was limited by better targeting. However, the impact of TMZ along with the methylation status of the O-6-methylguanine-DNA methyltransferase (MGMT) on survival was not systematically evaluated in the present study. This study has a selection bias which necessitates prospective studies, as the patients with methylated MGMT or who were in better clinical condition were more likely to have received adjuvant TMZ chemotherapy. Nevertheless, the results of this study support our initial work and further establish the efficacy of this HS-IMRT regimen combined with TMZ.

Both single-fraction stereotactic radiosurgery and brachytherapy have been reported to have modest utility as palliative salvage interventions; however, both have been associated with high rates of re-operation because of the associated toxicity [24, 25]. Our initial experience with HS-IMRT used the 5- to 7-Gy fractions to a maximum of 35 Gy and reported an actuarial reoperation rate of 4.8% for radiation necrosis, providing additional support that this dose and fraction size is well tolerated. In our opinion, MET-PET/CT/MR image fusion could facilitate target volume delineation and normal tissue sparing, which might lead to an improved therapeutic ratio. MET-PET/CT/MR image fusion planning, which examines biological behavior in re-irradiation, seems not only to decrease the likelihood of geographical misses in target volume definition, but also to facilitate normal tissue sparing and toxicity reduction, especially when conformal treatment technique such as HS-IMRT is implemented.

## Conclusions

This is the first prospective study using biologic imaging to optimize HS-IMRT using MET-PET/CT/MRI image fusion in recurrent GBM. A low frequency of side effects was observed. HS-IMRT with PET data seems to be well tolerated and resulted in a median survival time of 11 months after HS-IMRT, although a properly designed randomized trial are necessary to firmly establish whether the present regimen is superior to the other treatment methods for recurrent GBM.
